# The relationship between endothelial dysfunction and dementia risk in Framingham Heart StudyQiushan Tao and Wendy Wei Qiao QiuBoston University School of Medicine

**DOI:** 10.1002/alz70857_103282

**Published:** 2025-12-25

**Authors:** Wendy Weiqiao Qiu

**Affiliations:** ^1^ Boston University School of Medicine, BOSTON, MA, USA; Boston University School of Medicine, 352 Beacon Street, Unit#5, MA, USA

## Abstract

**Background:**

Peripheral inflammation damages endothelia throughout the body, and increases Alzheimer's disease (AD) pathogenesis in the brain. Since brachial artery flow‐mediated dilation (FMD) evaluates conduit artery endothelial function, this study aimed to examine if FMD can be used to predict AD risk.

**Method:**

Data from 2844 individuals of the Framingham Heart Study (FHS) offspring cohort (Gen 2, Exam 7, 1998 ‐2024) includes baseline data on different variables of FMD, and incident dementia/AD, the measurements of plasma AD biomarkers and brain volumes. The average age at FMD measurements was 60.6 ± 9.4 (mean ± SD) years old, and 1512 (53.2%) of them were women.

**Result:**

The FMD measurements were collected at baseline and the main outcomes were AD dementia during the 17 years of follow‐up. Compared to those who remained normal cognitive function (*n* = 2325, 81.8%) had the highest levels of FMD and Hyperemic mean flow velocity followed by those who developed mild cognitive impairment (MCI) (*n* = 241, 8.5%), and those who developed dementia (*n* = 278, 9.8%) including 241 AD had the lowest levels of FMD (*p* <0.001) and Hyperemic mean flow velocity (*p* <0.001). After adjusting for age, sex, education, ApoE4 and different cardiovascular diseases, baseline endothelial function status including Flow‐mediated vasodilation (%) (HR, 95%CI: 0.83, 0.76‐0.91, *p* <0.001) as well as Hyperemic mean flow velocity (cm/s) (HR, 95%CI: 0.89, 0.79‐1.00, *p* = 0.049) were negatively associated with AD risk. Based on these endothelial statuses, we further divided the participants into three tertiles and found that the highest tertiles of all three variables (indicating healthy endothelia) had lower levels of plasma AD biomarkers and higher brain volumes than the lowest tertiles (indicating poor endothelial function).

**Conclusion:**

This is the first study to show the relationship between endothelial dysfunction and AD dementia risk in a community cohort study. The data further supports that healthy brain endothelia is a resilience mechanism during the aging process to protect from cognitive decline and AD dementia development.

